# Neurological update: structural and functional imaging in epilepsy surgery

**DOI:** 10.1007/s00415-023-11619-z

**Published:** 2023-02-15

**Authors:** Katie Yoganathan, Naveed Malek, Emma Torzillo, Menaka Paranathala, John Greene

**Affiliations:** 1 University of Oxford and Oxford University Hospitals, Oxford, UK; 2 Department of Neurology, National Hospital for Neurology and Neurosurgery, London, UK; 3 Department of Neurology, Queen’s Hospital, Romford, UK; 4 Department of Neurosurgery, Royal Victoria Infirmary, Newcastle, UK; 5 Department of Neurology, Institute of Neurological Sciences, Queen Elizabeth University Hospital, Glasgow, UK

**Keywords:** Epilepsy, SPECT, PET, Surgery, FMRI, EEG

## Abstract

Structural and functional imaging prior to surgery in drug-resistant focal epilepsy, has an important role to play alongside electroencephalography (EEG) techniques, in planning the surgical approach and predicting post-operative outcome. This paper reviews the role of structural and functional imaging of the brain, namely computed tomography (CT), magnetic resonance imaging (MRI), functional MRI (fMRI), single photon emission computed tomography (SPECT) and positron emission tomography (PET) imaging in the preoperative work-up of people with medically refractory epilepsy. In MRI-negative patients, the precise localisation of the epileptogenic zone may be established by demonstrating hypometabolism on PET imaging or hyperperfusion on SPECT imaging in the area surrounding the seizure focus. These imaging modalities are far less invasive than intracranial EEG, which is the gold standard but requires surgical placement of electrodes or recording grids. Even when intracranial EEG is needed, PET or SPECT imaging can assist in the planning of EEG electrode placement, due to its’ limited spatial sampling. Multimodal imaging techniques now allow the multidisciplinary epilepsy surgery team to identify and better characterise focal pathology, determine its’ relationship to eloquent areas of the brain and the degree of interconnectedness within both physiological and pathological networks, as well as improve planning and surgical outcomes for patients. This paper will update the reader on this whole field and provide them with a practical guide, to aid them in the selection of appropriate investigations, interpretation of the findings and facilitating patient discussions in individuals with drug-resistant focal epilepsy.

## Introduction

30–40% of people with epilepsy (PWE) will have drug-refractory disease that does not respond to treatment with anti-seizure medications [1]. All patients with drug-refractory focal epilepsy should be considered as potential candidates for resective surgery. 1.5% of people with newly diagnosed epilepsy may eventually require epilepsy surgery. Furthermore, there are many people with drug-refractory epilepsy who might benefit from epilepsy surgery but are not considered or assessed for this treatment option [2]. A detailed presurgical work-up is required to plan the surgical approach and technique, determine the chances of postoperative seizure freedom, and to minimise the risk of postoperative neuropsychological and functional impairment. The primary goal of this presurgical work-up is to identify the epileptogenic zone—the region or area of the brain generating the seizures, removal of which may render the patient seizure free [3], as well as localisation of higher functions. A range of clinical assessments, imaging and electrophysiological techniques can be used to identify the epileptogenic zone. The surgical work-up starts with a detailed review of seizure semiology, ictal and interictal electroencephalography (EEG) using video telemetry, and high-resolution magnetic resonance imaging (MRI) of the brain, and may include functional MRI (fMRI), particularly if there is the involvement of the dominant hemisphere or proximity to the primary motor, sensory or visual cortices.

In many cases, these investigations do not provide enough information to confidently proceed to surgery or may in fact show discordant results. In this subset of patients, functional imaging techniques such as positron emission tomography (PET) and/or single photon emission computerised tomography (SPECT), can help clinicians develop a clearer picture of the epileptogenic zone and eloquent areas, facilitating discussions around the role and aims of surgery and aid prognostication. Advanced techniques such as magnetoencephalography (MEG), electrical or magnetic source imaging (ESI/MSI), simultaneous EEG and fMRI, as well as automated imaging protocols for lesion detection, are being evaluated in research settings to gather additional information. Functional imaging may be used in select cases to determine where further invasive testing such as intracranial EEG may be helpful, or in some cases enable patients to proceed to direct surgical resection [4].

**Fig. 1:**
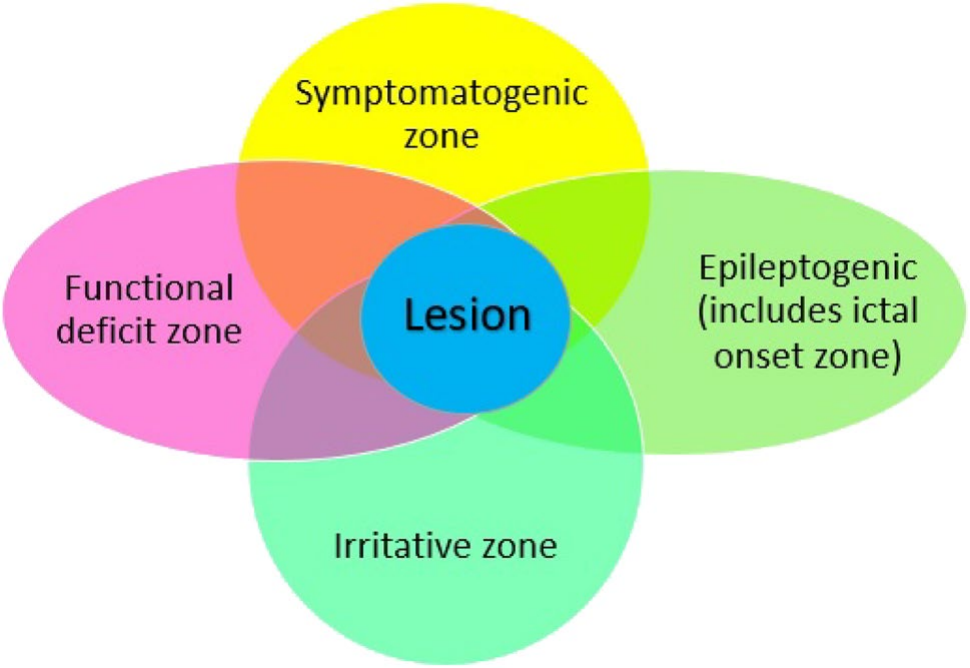
Cortical zones defined in the presurgical evaluation

Seizures are a dynamic process; brain states in an individual patient can vary dramatically during a seizure or the ‘ictal’ state, compared to the ‘interictal’ state representing the period between seizures. This means that the interpretation of imaging results must consider whether the patient is in an ictal or interictal state. Using functional imaging in the same patient in these two different states can be an invaluable tool for the clinician to better understand the physiological basis and the neural networks associated with epilepsy in an individual. The presurgical workup of PWE who are candidates for epilepsy surgery involves the identification of the symptomatogenic zone, the irritative zone, the ictal onset zone, the epileptogenic lesion, the epileptogenic zone and the eloquent cortex [5]. These theoretical regions can overlap or be anatomically distinct in individual patients [6, 7] (Fig. 1). Multiple modalities are used to define these regions. Functional imaging with PET can show both a focal region of abnormal metabolism associated with a lesion such as focal cortical dysplasia (FCD), but could also demonstrate a wider hypometabolic network, for example involving both frontal and temporal lobes, which can indicate the functional deficit zone. The functional deficit zone is the area of the cortex that is functionally abnormal in the interictal period. This area of abnormality can be detected by both PET and SPECT imaging. This deficit zone can be a direct consequence of the destructive effect of the lesion or may be functionally mediated as a result of abnormal neuronal transmission [5]. The aim of epilepsy surgery is the complete resection or disconnection of the epileptogenic zone, to render the patient free of disabling seizures (Table 1), whilst conserving eloquent regions and preventing unforeseen neuropsychological deficits [5].

**Table 1:** Engel classification of outcomes after epilepsy surgery [8]

Classification	Outcome
Class I	Free of disabling seizures
Class II	Rare disabling seizures
Class III	Worthwhile improvement
Class IV	No worthwhile improvement

This paper will focus on the role of functional imaging techniques of the brain, with an initial overview of structural imaging techniques, in the preoperative workup of people with medically refractory epilepsy.

## Structural brain imaging

### Computed tomography (CT)

In an emergency setting, when a patient has new onset seizures, a non-contrast CT of the head is appropriate to diagnose or rapidly exclude several life-threatening conditions such as an epidural haematoma or subarachnoid haemorrhage. MRI is more time consuming and, therefore, less appropriate for these pathologies in the acute setting [9]. In refractory epilepsy, CT scans can also be useful to identify calcifications associated with disorders such as neurocysticercosis, or to detect bone abnormalities related to temporal encephalocoeles, which are less well characterised on MRI. Even when an emergency department CT head scan is normal, an outpatient MRI head scan is required to clarify whether there is any underlying structural lesion that will predispose the person to further seizures in the future.

### Structural MRI

The presurgical workup of refractory epilepsy patients begins with structural imaging using MRI. This modality is more sensitive, more specific, has a higher spatial resolution than CT for subtle structural abnormalities, and can be used to acquire several different sequences. The optimal epilepsy surgery protocol includes axial and coronal slices of volumetric T1-weighted sequences for improved grey-white matter differentiation, detection of cortical thickness and malformations of cortical development. Fluid-attenuated inversion recovery (FLAIR) sequences are sensitive to pathologies such as hippocampal sclerosis (HS), FCD, tumours, inflammation, and scars. Quantification of hippocampal changes can also be assessed with volumetry and T2 relaxometry. T2* gradient echo or susceptibility weighted sequences are useful to detect calcified and vascular lesions, such as cavernomas and arteriovenous malformations. Voxel based morphometry (VBM) is a neuroimaging technique that involves a voxel-wise comparison of the local concentration of grey matter between two groups of subjects [10]. Although one study of 34 people with epilepsy showed there was poor agreement between video EEG and VBM (Cohen’s kappa = 0.099) compared to video EEG and PET scans (kappa > 0.4) [11], which calls into question its clinical utility.

As the primary role of MRI in epilepsy is in the detection of structural abnormalities, the quality and resolution of the images generated could determine the outcomes of epilepsy surgery [12]. A repeat MRI at higher resolution may uncover hitherto unidentified subtle lesions such as a small FCD, and this is particularly important when trying to identify an epileptogenic lesion in the brain. A 3 Tesla (3T) MRI or higher magnetic field strength MRI, is preferred over 1.5 Tesla (1.5T) scanners as it offers a higher resolution of the mesial temporal lobe and other structures [13]. This is due to the higher signal-to-noise ratio and its’ higher T2 contrast [12]. A 3T MRI also provides better resolution of structural details for co-registration with functional studies, such as PET [12]. Furthermore, in the case of MR spectroscopy, using a 3T machine for generating images with a higher chemical shift, increases both the quality as well the speed of acquisition [12], which makes it more tolerable for patients. Several centres are now using 7 Tesla (7T) MRI which, in conjunction with the optimisation of post-acquisition image processing, increases the diagnostic yield [14]. The type of MRI coil used can also make a difference; for instance, a phased array coil receives signal to produce an image from several coils rather than a single coil, thereby improving the signal-to-noise ratio and facilitating faster imaging [4].

Newer testing modalities in conjunction with 7T MRI include glutamate-weighted chemical exchange saturation transfer (GluCEST) for mapping hippocampal glutamate distribution in epilepsy, allowing researchers and clinicians to differentiate lesional from non-lesional mesial temporal lobe epilepsy (TLE) [15].

### Diffusion-weighted and diffusion tensor MR imaging

Diffusion-weighted imaging (DWI) and diffusion tensor imaging (DTI) are MRI sequences that are highly sensitive to the diffusion of water molecules [16]. When diffusion is limited, such as in white matter tracts (WMT), the movement becomes forced in a specific direction. This data can be used for the reconstruction of WMT, in a process known as tractography. Diffusion tensor tractography (DTT) studies have shown that poorer postsurgical outcomes are associated with the tract density of certain white matter segments in TLE [17]. This has the potential to aid in the delineation of disrupted WMT and act as a prognostic marker [17]. As DTT enables the non-invasive in-vivo delineation of WMT, it is also useful to identify eloquent white matter connections such as the optic radiation [4].

DTI can also better inform us about radiographic diagnoses such as mesial temporal sclerosis (MTS), the most common cause of TLE [18]. MTS patients show lower orientation dispersion indices and elevated axial diffusivity in the dentate gyrus on DTI compared to no MTS [18]. DWI is more useful in determining the extent and severity of early neuronal damage caused by epileptic discharges in status epilepticus patients [19], but is not particularly useful in the workup for epilepsy surgery.

Diffusion kurtosis imaging (DKI) is an emerging technique, extending DTI’s capabilities, enabling the characterisation of non-Gaussian water diffusion behaviour [20], and is considered sensitive for the detection of diffusion abnormalities in both white matter and grey matter of idiopathic generalised epilepsy in children [21].

## Functional brain imaging

Functional imaging techniques broadly investigate dynamic processes of metabolism, perfusion, or gas exchange to identify abnormal areas, as opposed to structural imaging which demonstrates anatomical static abnormalities.

### Functional MRI (fMRI)

FMRI is mostly utilised in the presurgical evaluation of patients with drug-resistant epilepsy to localise eloquent brain areas such as language and motor cortices. This enables planning of surgery to minimise any damage to these areas, and to reduce postsurgical language or motor impairments [22].

FMRI uses the diamagnetic and paramagnetic properties of oxygenated and deoxygenated blood respectively, to produce signals by measuring their relative ability to induce local magnetic field distortions. This so-called ‘blood oxygen level dependent’ (BOLD) response alters in conjunction with an increase in local neuronal activity and, therefore, is regarded as an indirect measure of local neuronal activity [23].

To investigate motor function, self-triggered movements are commonly used. These include tongue, finger, and toe movements, contralateral to the side of the lesion, to localise the motor homunculus in relation to the lesion. The somatosensory functional areas can also be studied using tactile stimuli. Language functions are examined using various paradigms involving auditory or visual stimulation, such as the “verbal fluency task” for anterior (mainly frontal lobe) language areas, and “semantic decision-making task” for more widely distributed networks including anterior and posterior language areas [24]. In temporal lobe epilepsy, auditory and picture naming tasks are useful to assess posterior temporal lobe function, with stronger activations correlating with better clinical naming scores [25].

More invasive techniques for localisation of functions include direct cortical stimulation (DCS), which involves testing of motor, sensory, or higher level neurocognitive functions with stimulus presentation, coordinated with the administration of electrical stimulation to the exposed brain following craniotomy, to localise functions [26]. Disadvantages of DCS are the surgical risk of the procedure, discomfort, extended hospital stay and limited cortical coverage [27]. Another test is the Wada test which involves intracarotid amytal injection to anaesthetise each cerebral hemisphere in turn, after which the capacity of each hemisphere to support language and memory function can then be assessed [28].

FMRI appears to be a promising alternative to invasive tests such as DCS or the Wada test, particularly in cases where the invasive localisation of the eloquent cortex has proven to be challenging but more importantly because of the much lower morbidity of the procedure itself [29]. Wada test has been superseded by fMRI in the United Kingdom and other countries over the last decade in centres where fMRI technology is readily available, and it is considered of historical importance only. In addition, when compared to the Wada test, fMRI is less costly, may be repeated if necessary and can provide information about intra-hemispheric localisation as well as lateralisation of language process, which the Wada test is not able to do. This is dependent on the threshold used, as regions activated on fMRI images tend to be more extensive than those identified on cortical stimulation mapping. These activated areas may not be crucial to language functions, which could lead to more conservative surgical decisions. Likewise, if the threshold is too stringent, non-activated areas may still be important in language. Studies have shown combining fMRI and cortical stimulation mapping may predict language outcomes after epilepsy surgery better than either test alone [27]. FMRI sensitivity and specificity, compared to DCS were 80.6% and 72.7% in one study [30] and when fMRI and DCS language findings were concordant, the combined tests showed a sensitivity of 100% and a specificity of 75% for language outcome post-resective surgery [27]. Therefore, fMRI could be a useful predictor of postoperative deficits, and can help in mitigating risks of resective surgery by the preservation of areas of eloquent cortex [14]. Overall, fMRI can provide a more thorough picture of the complexity of a patient’s language organisation in the brain, and infer and modify the procedural risk of future surgery [31].

### PET imaging in Epilepsy

Interictal PET or ictal SPECT have been established as useful investigations to localise the origins of focal epilepsies. These modalities can be used to predict outcomes in selected epilepsy patients proceeding to surgical resection, particularly for those with normal MRI or discordance between MRI, clinical and EEG data.

Ictal and interictal patterns of local cerebral metabolic rate have been studied in PWE using PET with 18F-labeled 2-fluoro-2-deoxy-D-glucose (18F-FDG) since the 1980s. PET (specifically with 18F FDG) reflects the rate of glucose uptake in the brain parenchyma (which is an indirect measure of neuronal function), while SPECT represents cerebral blood flow changes [32]. Glucose metabolism is reduced in abnormal tissue and increased during ictal activity. Ictal increases and decreases in local cerebral glucose metabolism are observed in different seizure types. During generalised tonic–clonic seizures, a diffuse ictal increase and post-ictal decrease in cerebral metabolism is observed. Absence seizures are associated with a diffuse increase in cerebral metabolic rate. On the other hand, focal-onset seizures are associated with the activation of specific brain regions unique to each patient, reflecting the area of abnormal electrical discharge in the brain. These focal area(s) typically show decreased cerebral metabolism on PET imaging during the interictal phase [33]. Ictal PET is not as well studied, due to the difficulties in practically arranging and performing a PET scan during a seizure. In addition, FDG uptake takes around 30–45 min duration, so is difficult to obtain during an ictal period, which is typically between 30 seconds to 2 minutes. However, it has been used in patients with focal status epilepticus, with prolonged seizures lasting greater than 30 minutes, and this has revealed a region of hypermetabolism in a number of patients, correlating with the likely ictal onset zone [34].

#### Interpretation of PET imaging data

Both visual PET analysis and quantitative PET (qPET) analysis predict outcome after temporal lobectomy, although quantitative measures offer more precise information [35]. In qPET imaging, a side-to-side percent asymmetry index (AI) of uptake values within specific regions of interest (ROI) are calculated. The AIs are calculated as: [(mean value ROI – mean value contralateral ROI)/ (mean value ROI + mean value contralateral ROI) × 100], for lateral temporal, mesial temporal, parietal, inferior frontal, and superior frontal regions. Patients are considered to have unilateral temporal hypometabolism, if the mesial or lateral temporal AI > 10–15% is present, or if greater than 2 standard deviations from the mean of normative data [36, 37]. The mechanisms involved in the generation of a seizure that precedes the 18F FDG scan appear to influence the interictal hypometabolic pattern seen on PET imaging. Therefore, caution must be used when interpreting scans that are preceded by a non-habitual seizure [17].

There are several patterns of PET hypometabolism that have now been associated with distinct types of focal epilepsies, and in some cases have been shown to predict post-surgical outcomes [27]. In TLE patients, PET displays a high sensitivity in localising the seizure focus (range 71–89%). The sensitivity is increased further when PET results are combined with other modalities (MRI and/or EEG) [38].

#### PET imaging in TLE

TLE is the most common type of focal epilepsy, and with careful investigation and preoperative planning, it is associated with the best outcomes following epilepsy surgery. TLE patients show asymmetries in FDG uptake which may be due to mild hypermetabolism or mild hypometabolism in the epileptic temporal lobe [39]. Patients with an electroclinical syndrome consistent with mesial TLE will often have evidence of hippocampal sclerosis (HS) on MRI. However, a significant number of these patients with clinical and EEG features of temporal lobe seizures have no evidence of HS on MRI, yet many of these patients have concordant hypometabolism of the temporal lobe on 18F-FDG PET [40]. These HS-negative but PET-positive TLE patients represent a surgically remediable group distinct from HS TLE patients, with a pathophysiological basis that primarily involves epileptic circuits in the lateral temporal neocortex rather than mesial temporal structures [41].

TLE with amygdala enlargement probably represents a distinct nosological and less homogeneous syndrome without ipsilateral HS. Chronic inflammatory processes in the temporal lobe or an amygdala FCD could possibly lead to amygdala enlargement. Interictal 18F-FDG PET shows regional glucose hypometabolism in the ipsilateral temporal lobe in these cases in concordance with EEG and MRI data [42]. The underlying pathology in some of these cases has been proven to be neoplastic (low-grade astrocytoma and gangliocytoma) but non-neoplastic lesions have also been documented. In patients with non-neoplastic lesions (FCD, vacuolar degeneration, and hamartoma) causing amygdala enlargement, there is no increased uptake on PET scanning. Thus, increased radiotracer uptake in the enlarged amygdala may provide some information for selecting surgical candidates who may require immediate attention [43].

More extensive patterns of PET hypometabolism can also be seen in TLE patients, including extra-temporal and frontal lobes [44], which may explain some of the executive dysfunction and working memory deficits noted on neuropsychological testing in these patients [45]. Studies have also shown in patients with widespread glucose hypometabolism, epilepsy surgery may not result in complete seizure freedom despite the complete removal of the MRI-identified lesion. This data can be valuable in counselling the patient preoperatively. The volume of significant glucose hypometabolism remote to the ictal-onset zone may be an independent predictor of the success of epilepsy surgery in rendering the patient seizure free [46].

Whilst FDG-PET is used in clinical practice for epilepsy surgery, in research studies using PET with specific ligands, one is able to probe the neurochemistry associated with the pathophysiology of epilepsy [14]. AMPA (α-amino-3-hydroxy-5-methyl-4-isoxazole propionic acid) receptors are thought to have a role in the pathophysiology of epilepsy. One exploratory study developed a novel PET tracer labelled with [^11^C]K-2 that specifically bound in vivo to AMPA receptors, and found increased [^11^C]K-2 uptake in the epileptogenic focus of patients with mesial TLE, which was closely correlated with the local AMPA receptor protein distribution in surgical specimens resected from these same individuals [47].

#### PET imaging in extra-temporal epilepsy

PET findings in extra-temporal lobe epilepsies (ETLE) are diverse, due to the broad range of underlying aetiologies. An important cause of ETLE is FCD. PET may provide a complimentary role in recognising and defining these often subtle lesions through co-registration with MRI [48]. The inclusion of 18-F FDG PET/MRI co-registration as part of the multimodality presurgical evaluation can enhance the non-invasive identification and successful surgical treatment of patients with FCD, especially for the third of patients with discordant findings and those with ‘normal’ 1.5T MRI scans due to cortical dysplasias with only subtle radiological findings [49]. However, broader patterns of temporal glucose hypometabolism are not uncommon in ETLE, thus, interpretation of interictal FDG-PET results in ETLE requires consideration of EEG results and seizure semiology to avoid false localisation particularly in non-lesional epilepsy [50].

### SPECT imaging in epilepsy

In contrast to PET imaging which detects changes in local cerebral metabolic rates, SPECT imaging commonly uses Technetium-99-based radiotracers to detect changes in cerebral perfusion in areas surrounding the epileptogenic zone. This is based on the concept of cerebral metabolic and perfusion coupling, which is that an increase in neuronal metabolic activity in the seizure propagating regions is associated with an increase in CBF or hyperperfusion in those areas [51]. Cerebral perfusion is linked to neuronal activity, so it is used as a marker of abnormally reduced (interictal) or increased (ictal) neuronal activity.

Although SPECT imaging can be performed in the ictal, interictal or post-ictal period, the highest sensitivity for seizure localisation is in the ictal period [52]. Ictal SPECT sensitivity is 97% to 100%, followed by post-ictal SPECT sensitivity of 75–77%, while interictal SPECT has the lowest sensitivity of 43–44% [32]. Despite its’ poorer sensitivity and specificity, interictal SPECT is complimentary to the ictal SPECT, and allows qualitative visual comparison and quantitative subtraction using automated protocols [53]. Most centres will perform an ictal SPECT scan (showing focal hyperperfusion) with an interictal SPECT scan (showing focal hypoperfusion) and concordance between these regions enhances the specificity of the diagnostic test [54]. Post-ictal SPECT scans are not routinely used in clinical practice, and when performed, are more likely due to the “ictal” injection inadvertently being administered after the seizure has terminated.

#### Interpretation of SPECT imaging data

Raw data obtained from a SPECT scanner is smoothed using a Butterworth filter. The goal of filtering is to compensate for the loss of detail in an image while reducing noise [55]. Attenuation (scattering and absorption) of gamma photons in the patient’s body is one of the major limitations of SPECT imaging. It reduces the quantitative accuracy of measured radioactivity concentrations and causes hot rim artifacts in reconstructed images, if not corrected [56]. An attenuation correction such as Chang’s method is, therefore, applied and images are then reformatted in axial, coronal, and sagittal planes. Finally, the reformatted images are analysed using a graded colour scale. The cerebellum is used as a reference site (100% maximum value). Any decrease or increase in cerebral perfusion in the ROI compared with the cerebellum is considered abnormal using predefined cut-offs.

**Fig. 2:**
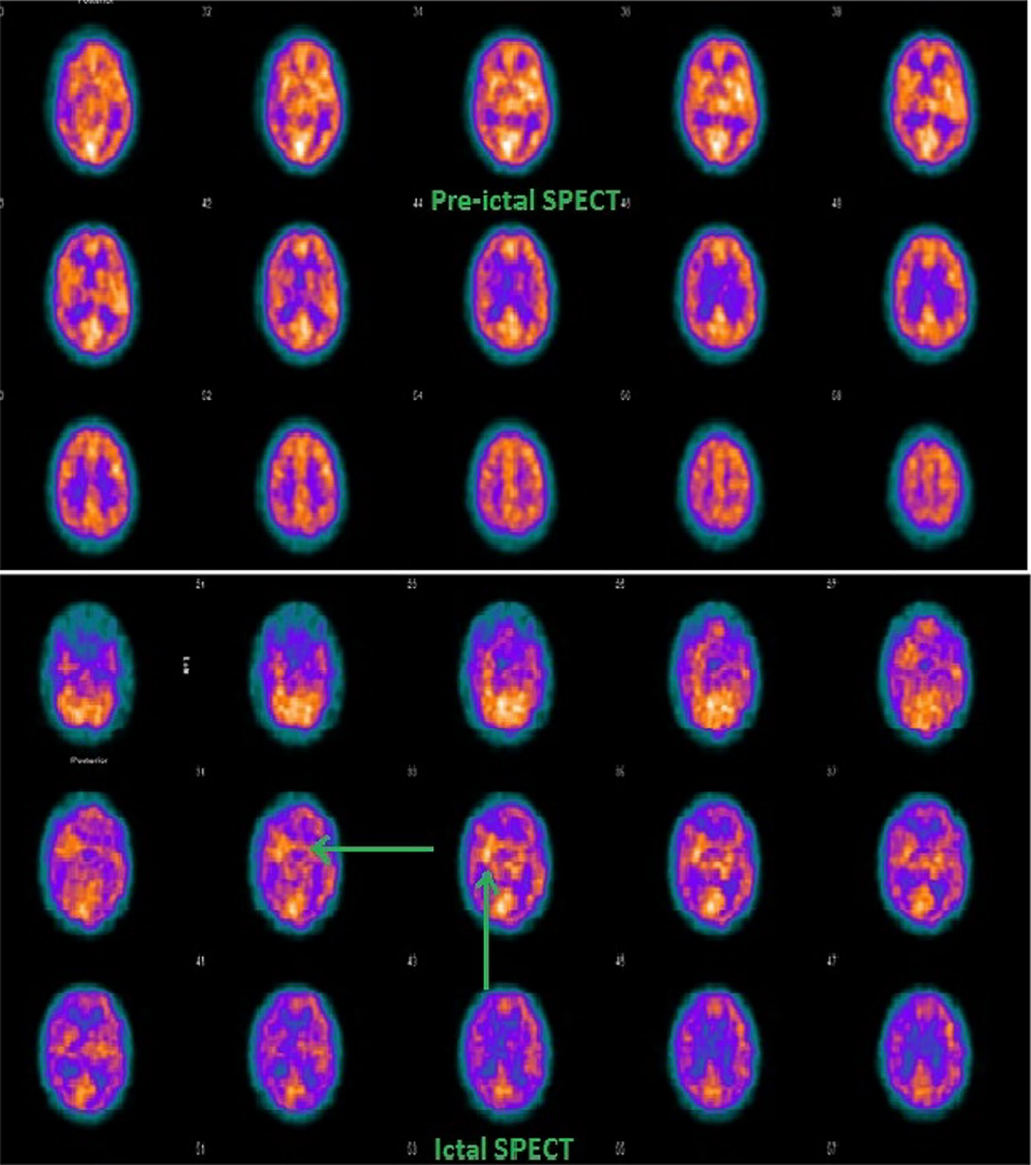
Tc-99 m HMPAO pre-ictal brain SPECT scan (top image) an ictal scan (bottom image) section showing a focal area of hyperperfusion in the right temporal area (green arrows) suggesting a locus of seizure onset here

Computer-aided image post-processing, with subtraction of ictal SPECT co-registered to MRI (SISCOM), can significantly improve the clinical usefulness of SPECT in localising the seizure focus prior to surgical resection [57] (Fig. 2). SISCOM has been demonstrated in a prospective study to alter the consensus decision-making process in epilepsy surgery [57]. However, SISCOM does not compensate for the physiologic variance in CBF that can show significant asymmetries in multiple areas of the brain. Statistical parametric mapping (SPM) can be used to overcome this by comparing perfusion changes in PWE to a control group without epilepsy [58]. SPM is an image-analysis tool that assesses the statistical significance of CBF changes on a voxel-by-voxel basis, thereby removing the subjectivity inherent in conventional ROI analysis [59].

The accuracy of SPECT in localising the seizure focus pre-operatively in patients with drug-refractory epilepsy has been found to be high when considering the final diagnosis reached by the convergence of clinical, electrophysiological, structural, pathological and outcome data, as discussed below.

#### SPECT imaging in TLE and ETLE

In a study of 75 consecutive patients with epilepsy (48 with TLE and 27 with ETLE), ictal SPECT correctly identified the epileptogenic zone in 91% of patients when the two study groups were analysed separately. The specificity and positive predictive values were greater than 90% for the whole series of patients. In up to two-thirds of these cases, ictal SPECT either obviated the need for invasive EEG, thus avoiding the associated risks, or helped to define where to concentrate the efforts of more invasive investigations [60].

Ictal SPECT has a lower localising value in ETLE (90%) compared to TLE (97%) [61]. This discrepancy may be explained by the fact many extra-temporal seizures are very brief and during that very short period, true ictal SPECT examinations can be difficult to achieve due to the need to administer the radiotracer at the onset of seizure [61].

## Comparison of PET and SPECT imaging

Both PET and SPECT imaging can be used to localise a seizure focus but PET has a better spatial resolution (2–3 mm) than SPECT. Local expertise and available technology usually determine the choice of modality used. SPECT scanning with CT is less expensive (tariff including the cost of reporting in the NHS is £102 [62] and more readily available than PET scanners in most hospitals in the United Kingdom. Of the two modalities, studies suggest that ictal subtraction SPECT is more sensitive than interictal PET [63].

Several smaller head-to-head comparisons of PET and SPECT in localising a seizure focus have been performed. In a study of 45 patients who had intracranial EEG monitoring to confirm the seizure onset zone, PET scans identified the same region in 25 cases (56% sensitivity) and SPECT in 39 cases (87% sensitivity) [63]. In another study of patients in whom both MR imaging and FDG-PET failed to localise the epileptogenic zone, ictal SPECT was associated with post-surgical seizure freedom in 86% of patients with histologically proven FCD in whom the zone of ictal hyperperfusion detected on SPECT was completely resected [64]. However, there is a trade-off between increased sensitivity of SPECT and the area of abnormality identified on SPECT. Seizures produce early dynamic perfusion patterns, resulting in hypoperfusion and hyperperfusion, likely due to the propagation of epileptic activity and initiation of inhibitory mechanisms [65]. The use of perfusion SPECT imaging allows the investigation of ictal activations, but the low temporal resolution of ictal perfusion SPECT often results in the detection of both the ictal onset zone as well as the seizure propagation pathways so the area may be larger than the ictal zone. The full propagation seizure pathway is not an area that always needs to be resected to render a patient seizure free [66] and this should be borne in mind when planning surgery so as to minimise the risk of neurological deficits.

## Combining different imaging modalities for localising the epileptogenic zone

Researchers have endeavoured to define an optimal battery of imaging modalities for presurgical localisation of the epileptogenic zone in MR-negative focal epilepsies [67]. Quadrimodal imaging procedures involving a hybrid PET/MR scanner (for single-session FDG-PET and MRI) and simultaneous high-density EEG-fMRI (256 electrodes), in a single session has been used to minimise scanning and recording sessions. This is associated with less radiation exposure than PET/CT. Moreover, this quadrimodal imaging platform provides the same medication level (anti-seizure medications) and biological conditions for all the modalities (PET, MRI, EEG, fMRI). Not surprisingly, the procedure is reported to improve workflow, reduce the duration and cost of presurgical epilepsy evaluation [68]. However, at present this approach is not widely available. There are technical concerns, and large-scale studies performed using this approach are limited by their retrospective nature, warranting prospectively designed studies to evaluate this combination of investigations further [69]. In addition, complicated protocols combining all modalities for all patients, instead of using a tailored approach in individual cases, can be time consuming and when squeezed into a short time window may lead to compromises in the quality of reporting and data analysis.

Quantitative PET (qPET) has a higher sensitivity than morphometric analysis programs (MAP; an MRI post-processing imaging analysis technique) of 73% versus 64% in MRI-negative epilepsy patients. However, combining MAP and qPET from a simultaneous PET/MRI scanner has higher specificity (88%) compared to either MAP (69%) or qPET (65%) alone. Any combination that can improve sensitivity and specificity would be valuable in aiming for Engel Class 1 outcomes post-resective surgery [70] but this may not be so useful in ETLE [71]. Furthermore, metabolic connectivity assessed by seed correlation analysis is associated with prognostic outcome in surgically treated TLE. A strengthened epileptogenic connectome is seen in patients with surgical outcomes other than Engel Class 1A (completely seizure-free since surgery) [72].

## Outcomes of image-guided localisation prior to surgery

A meta-analysis of 21 studies involving almost 1,200 patients undergoing epilepsy surgery showed an overall rate of post-operative seizure freedom (Engel Class I outcome) of 45.1% [73]. One of the significant predictors of long-term seizure freedom was abnormal preoperative MRI (RR 1.64, 95% CI 1.32–2.08) [73], confirming the importance of high-quality structural imaging in the evaluation of patients prior to epilepsy surgery. PET and SPECT functional imaging techniques are useful in presurgical decision-making as they can aid in identifying the epileptogenic zone in previously ‘MRI negative’ patients and can also help guide planning for intracranial EEG recordings by confirming laterality and highlighting other adjacent or distant regions likely to be involved in the epileptic network. The concordance of findings from intracranial EEG monitoring and ictal SPECT can lead to an Engel class 1 outcome (Table 1) in up to two-thirds of patients (66%) undergoing surgical resection but only half in those with intracranial EEG and PET concordance (50%) [63]. Unilateral hypometabolism on FDG-PET scans prior to surgery is predictive of good outcomes particularly in TLE with non-lesional MRI or equivocal ictal EEG recording, whereas bilateral hypometabolism on FDG-PET has a less favourable surgical outcome [53]. A meta-analysis of 46 studies showed that ipsilateral PET hypometabolism had a predictive value of 86% for good outcome post-resective surgery [74]. The positive predictive value was 80% in patients with a normal MRI and 72% in those with non-localised ictal scalp EEG [74].

While PET or SPECT may enable one to proceed to direct surgical resection in some cases, it is now recognised that the generators of epilepsy in individual patients may extend beyond an identified lesion, or involve highly connected, distant regions of the brain [75]. Intracranial EEG monitoring is therefore considered the gold standard in localising and defining the epileptogenic zone and may still be required in many cases prior to surgery. Functional imaging can however provide valuable information to allow the planning of intracranial EEG recording, which has very limited spatial sampling so that a detailed hypothesis must be established prior to implantation.

## Artificial intelligence and machine learning workup prior to surgery

Automated imaging analysis for presurgical planning and prediction of epilepsy surgical outcomes using machine learning techniques are being investigated [76]. With increasing computational capabilities and availability of effective machine learning algorithms validated and trained on large datasets, researchers are studying their role in daily clinical practice involving the medical and surgical management of PWE [76]. A study found postprocessing analysis of MRI data with automated machine learning algorithms was able to detect subtle structural abnormalities in MRI-negative patients [77]. Similarly, a software-based statistical analysis of PET and SPECT (STATISCOM] was superior to qualitative analysis alone in identifying focal abnormalities in MRI-negative patients [78]. A combined surface analysis of MRI and FDG-PET by a machine-learning approach detects FCDs in a higher proportion of patients (93%) compared to manual lesion labelling approaches (68–82%) [79]. This is not yet used widely in clinical practice but it is a growing area of interest [80].

## Conclusion and future directions

Functional imaging for localising the seizure focus in drug refractory epilepsy, particularly in those with no structural lesion identified on MRI, plays an important role in the workup of PWE. It enables the highest chance of being rendered seizure-free by surgery and minimises risks to eloquent areas. The techniques vary from fMRI to PET and SPECT.

Other emerging imaging techniques such arterial spin labelling (ASL) are capable of quantifying local cerebral blood flow by measuring the inflow of magnetically labelled arterial blood into the target region. Pulsed ASL may be a promising alternative to PET imaging for the detection of interictal hypoperfusion in TLE, due to its’ relatively easy accessibility, in places where PET imaging is not available. This would be practically very useful for patients in smaller hospitals if its’ sensitivity was similar to PET; data from larger studies is still needed though to validate this finding.

Further research is required to evaluate higher-resolution MRI and identify radiotracers and ligands for PET and SPECT imaging with higher sensitivity, particularly for extra-temporal epilepsy. Using PET with specific ligands could help us understand the pathophysiology of seizures, and improve the future management of epilepsy patients. Looking to the future, artificial intelligence and machine learning approaches hold promise for improving localisation and surgical outcomes (Table 2).

**Table 2:** Key points

1. Functional imaging uses changes in cerebral metabolism and perfusion to identify eloquent, and abnormal regions of brain tissue or neuronal activity and aids decision-making in epilepsy surgery.
2. FMRI uses the magnetic properties of oxygenated and deoxygenated blood to assess dynamic changes in neuronal tissue during language or motor tasks.
3. SPECT imaging detects cerebral perfusion changes, and during a seizure the abnormal, synchronised neuronal activity can be identified using ictal SPECT, due to the close relationship between electrical activity and blood flow.
4. PET imaging detects glucose metabolism and is typically performed interictally to measure abnormal regions of glucose metabolism associated with pathological brain tissue, which may generate or be due to seizures.
5. Computerised algorithms and software can be used to provide quantitative and co-registered analysis of high-resolution fMRI, PET, and SPECT data.
6. Artificial intelligence and machine learning approaches are being developed to analyse data from structural and functional imaging, to localise epileptogenic foci prior to resection surgery.

## Data Availability

Data sharing is not applicable to this article as no new datasets were created or analysed in this study.
